# Protein Tyrosine Phosphatases: Regulators of CD4 T Cells in Inflammatory Bowel Disease

**DOI:** 10.3389/fimmu.2018.02504

**Published:** 2018-10-31

**Authors:** Kelly A. Pike, Michel L. Tremblay

**Affiliations:** ^1^Department of Microbiology and Immunology, McGill University, Montréal, QC, Canada; ^2^Inception Sciences Canada, Montréal, QC, Canada; ^3^Rosalind and Morris Goodman Cancer Centre, McGill University, Montréal, QC, Canada; ^4^Division of Experimental Medicine, Department of Medicine, McGill University, Montréal, QC, Canada; ^5^Department of Biochemistry, McGill University, Montréal, QC, Canada

**Keywords:** protein tyrosine phosphatase, CD4 T cells, cytokine, JAK-STAT, inflammatory bowel disease

## Abstract

Protein tyrosine phosphatases (PTPs) play a critical role in co-ordinating the signaling networks that maintain lymphocyte homeostasis and direct lymphocyte activation. By dephosphorylating tyrosine residues, PTPs have been shown to modulate enzyme activity and both mediate and disrupt protein-protein interactions. Through these molecular mechanisms, PTPs ultimately impact lymphocyte responses to environmental cues such as inflammatory cytokines and chemokines, as well as antigenic stimulation. Mouse models of acute and chronic intestinal inflammation have been shown to be exacerbated in the absence of PTPs such as PTPN2 and PTPN22. This increase in disease severity is due in part to hyper-activation of lymphocytes in the absence of PTP activity. In accordance, human PTPs have been linked to intestinal inflammation. Genome wide association studies (GWAS) identified several PTPs within risk loci for inflammatory bowel disease (IBD). Therapeutically targeting PTP substrates and their associated signaling pathways, such as those implicated in CD4^+^ T cell responses, has demonstrated clinical efficacy. The current review focuses on the role of PTPs in controlling CD4^+^ T cell activity in the intestinal mucosa and how disruption of PTP activity in CD4^+^ T cells can contribute to intestinal inflammation.

## Introduction

**Figure 1:**
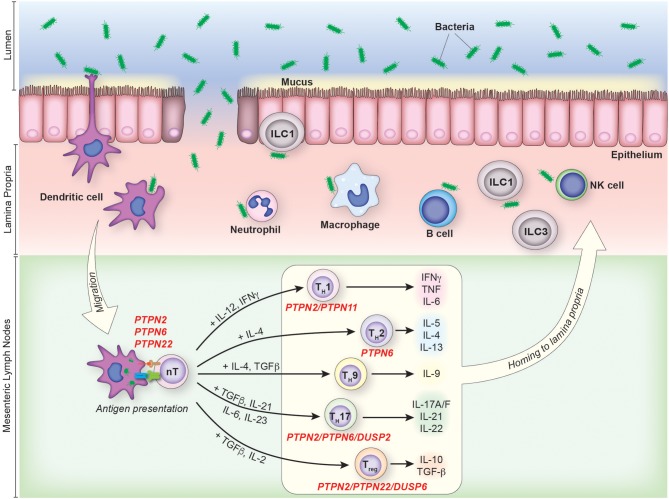
The contribution of PTPs to CD4^+^ T cell activation and differentiation in colonic inflammation. IBD pathophysiology is associated with the disruption of the intestinal mucosal barrier due to genetic, environmental and/or immunological factors. Under such circumstances, an increase in the uptake and processing of luminal antigens by innate immune cells initiates and maintains the chronic inflammatory response characteristic of IBD. CD4^+^ T cells are activated in the mesenteric lymph nodes following recognition of their cognate antigen presented in the context of MHC on the surface of antigen presenting cells. Activated CD4^+^ T cells then enter the lamina propria from circulation and perpetuate inflammation, secreting pathogenic pro-inflammatory mediators and chemokines which recruit additional leukocytes. PTPs involved in the activation and/or differentiation of specific T cell subsets are indicated.

The gastrointestinal track is a large mucosal surface at which the host's immune system is juxtaposed with a dense microbial population and a diverse array of dietary antigens. Immune recognition of enteric antigens however, is minimized by physical compartmentalization. Bacteria and dietary products are retained within the gut lumen, while the host immune system is localized in the mucosal tissue. This physical separation is preserved by the intestinal mucosal barrier, which includes a mucin layer, a single epithelial cell lining sealed by tight and adherens junctions and a continuous secretion of anti-inflammatory soluble mediators (Figure 1) (1–5).

The mucosal barrier is not absolute however, and a small number of bacteria do translocate from the lumen to the underlying lamina propria. In such instances, a complex network of innate and adaptive immune cells impedes the spread of intestinal bacteria in a manner that limits tissue damage (6, 7). This cellular network includes dendritic cells, resident macrophages and the largest population of T cells in the body.

Physical compartmentalization and mucosal immunity therefore establish and maintain microbiome-host mutualism (8). The loss of mutualism and a hyper-activation of the innate and adaptive immune system can result in intestinal inflammatory diseases such as Crohn's disease (CD) and ulcerative colitis (UC), the two main forms of inflammatory bowel disease (IBD). UC and CD are polygenic diseases characterized by chronic relapsing inflammation that results in intestinal pain, intestinal bleeding and diarrhea (9–11). While UC is restricted to the large intestine where it manifests as a uniform continuous pattern of inflammation, CD can occur anywhere throughout the gastrointestinal track in patches. The thickness of the inflammation also distinguishes the two diseases, UC being confined to the mucosa and CD presenting in both the mucosa and underlying muscle tissue (transmural).

The mechanisms that initiate and sustain IBD are incompletely understood. Current evidence supports a model in which genetic alterations and environmental factors, increase IBD susceptibility by deregulating the interplay between the microbiome, the intestinal epithelial barrier and the immune system. While the nature of pre-disposing environmental factors remains under debate, functional annotation of IBD-associated genes has identified gene variants that impact processes such as intestinal barrier function, anti-microbial activity, and autophagy (12–14). In addition, the disruption of the adaptive immune system has been implicated, with multiple IBD susceptibility genes being shown to contribute to CD4^+^ T cell development and function. Examples include *IL23R, JAK2*, and *STAT3* (12, 15–21).

### CD4^+^ T cells and IBD

CD4^+^ T cells direct suitable immune responses, maintain immune tolerance and support the differentiation of endurable immunological memory. However, CD4^+^ T cell subsets have also been shown to contribute to chronic intestinal inflammation, accumulating in the mucosa of both UC and CD patients (22). Additional evidence supporting a role for CD4^+^ T cells in IBD, is based on HIV^+^ IBD patients who, with a reduced total CD4 T cell count, have a higher incidence of remission as compared to non-HIV IBD patients (23, 24). Therapeutically, CD4^+^ T cell-depleting and blocking antibodies (cM-T412, MAX.16H5, and B-F5) have been shown to induce remission in both CD and UC patients (25, 26), while alternate therapies that inhibit the differentiation of CD4^+^ T cell subsets and the cytokines they secrete, have proven to be efficacious in IBD patients, These would include Tofacitinib (oral JAK inhibitor), Ustekinumab (human monoclonal antibody directed against IL-12 and Il-23) and Infliximab (chimeric hiamn/mouse monoclonal antibody directed against TNFα) (27–33). It should be noted, that such therapies also target other immune cell lineages and as such, efficacy may not be solely driven through a CD4^+^ T cell specific mechanism.

CD4^+^ T cells are classified into distinct subsets based on their inducing cytokines, transcription factor expression, and effector cytokine secretion. The initial classification of CD4^+^ T cells as T_H_1 IFNγ producers vs. T_H_2 IL-4 producers, has been broadened to include multiple additional subsets (34, 35). These subsets, and the cytokines they secrete, include T_H_9 (IL-9), T_H_17 (IL-17A, IL-17F, and IL-22), T_H_22 (IL-22), T follicular helper T_FH_ (IL-21) cells, as well as thymic-derived and peripherally-induced T regulatory cells (IL-10, TGFβ) (36–40) (Figure 1).

The contribution of the various CD4^+^ T cell subsets to CD and UC remains an area of ongoing research. Originally, CD was thought to be driven by T_H_1 T cells and UC by T_H_2 T cells. The use of such a T_H_1/T_H_2 paradigm to describe the different T cell responses involved in CD and UC has proven over simplistic however. It did not account for the role of more recently identified subsets such as T_H_17 T cells and Tregs. Moreover, the recent discovery of ongoing T cell plasticity in the intestinal mucosa of both CD and UC patients, has added further complexity to the CD4^+^ T cell response in these diseases (41, 42).

### Protein phosphorylation and CD4^+^ T cell differentiation

Protein tyrosine phosphorylation is required for CD4^+^ T cell differentiation and activation. Cascades of reversible protein phosphorylation events downstream of cytokine receptors (CytR), co-stimulatory molecules, and the T cell receptor (TCR), converge to induce gene expression profiles that drive CD4^+^ T cell activation and differentiation into distinct subsets (40).

**Figure 2:**
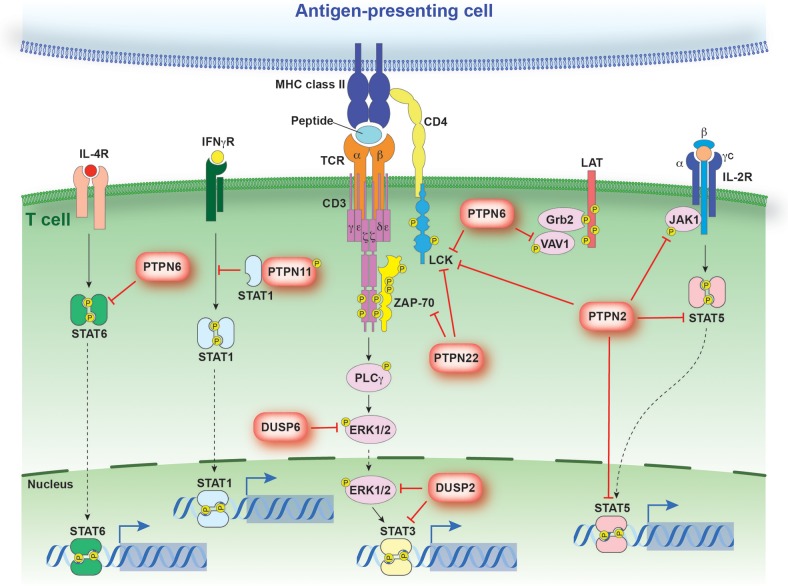
PTP regulation of antigen and cytokine receptor signaling. Schematic representation of signaling events regulated by PTPs discussed in the text. PTPs are linked to their respective substrates by a red bar-headed line. Dotted arrows depict translocation while solid black lines identify molecules linked in a signaling cascade. The direct interaction between STAT1 and PTPN11 models the sequestration of STAT1 from the IFNγR.

Naive T cells in peripheral circulation are activated upon TCR recognition of its cognate antigen in the context of major histocompatibility complex (MHC) expressed on antigen presenting cells. Upon TCR engagement, Src-family kinases (Lck, Fyn) are activated and phosphorylate tyrosine residues within the immune-receptor tyrosine-based activation motifs (ITAMs) in the TCR-associated CD3 and zeta chains (43–46). Phosphorylated ITAMs then provide docking sites for the recruitment and activation of the zeta-associated protein kinase (ZAP-70) (47). Cooperatively, Src-family kinases and Zap70 phosphorylate downstream signaling pathways which dictate the cellular response (Figure 2).

The strength of TCR signaling has a direct impact on CD4^+^ T cell differentiation (48). For example, Foxp3^+^ peripheral T regulatory (Treg) cells are generated primarily from CD4^+^ Foxp3^−^ T cells exposed to antigen under tolerogenic conditions or during homeostatic proliferation (49–52). In the presence of TGFβ, IL-2 and co-stimulatory signaling, intermediate TCR signaling induces Foxp3 expression and Treg differentiation (iTreg). By comparison, weak and strong TCR signaling are less potent at inducing Foxp3 expression (53, 54). Regulatory mechanisms are therefore required to establish and maintain the strength of TCR signaling within a given range. Such regulatory mechanisms include the modulation of protein tyrosine phosphorylation.

Critically, engagement of the TCR and co-stimulatory molecules is not sufficient to drive the polarization of CD4^+^ T cells (40). Rather, the presence of inductive cytokines and the activation of the Janus kinase (JAK)–signal transducer and activator of transcription (STAT) pathway is also required. Binding of cytokines to their corresponding receptor results in receptor dimerization, allowing for the juxtaposition and subsequent cross-phosphorylation of associated JAK molecules (55). Activated JAK molecules are then responsible for the phosphorylation of STAT family members. Tyrosine phosphorylated STAT molecules dimerize and translocate to the nucleus, where they promote specific gene expression profiles (Figure 2) (56–62).

Thus a network of signaling pathways, heavily dependent on tyrosine phosphorylation, directs CD4^+^ T cell activation and differentiation. The current review will examine the role of protein tyrosine phosphatases (PTP) in safeguarding this network, and how PTP deletion can perturb CD4^+^ T cell function and consequently contribute to intestinal inflammation.

### The protein tyrosine phosphatase family

The PTP family comprises a heterogeneous set of enzymes that were first defined by Tonks and colleagues by their capacity to dephosphorylate phospho-tyrosine residues and by their structurally related phosphatase catalytic domain (63, 64). PTP1B was the first phosphatase identified. Its sequence homology with a segment of the CD45 receptor protein (65), pointed to the existence of a conserved catalytic domain that became the main feature of the PTP gene family. CD45 also brought an interesting first link between PTPs and the immune system.

**Figure 3:**
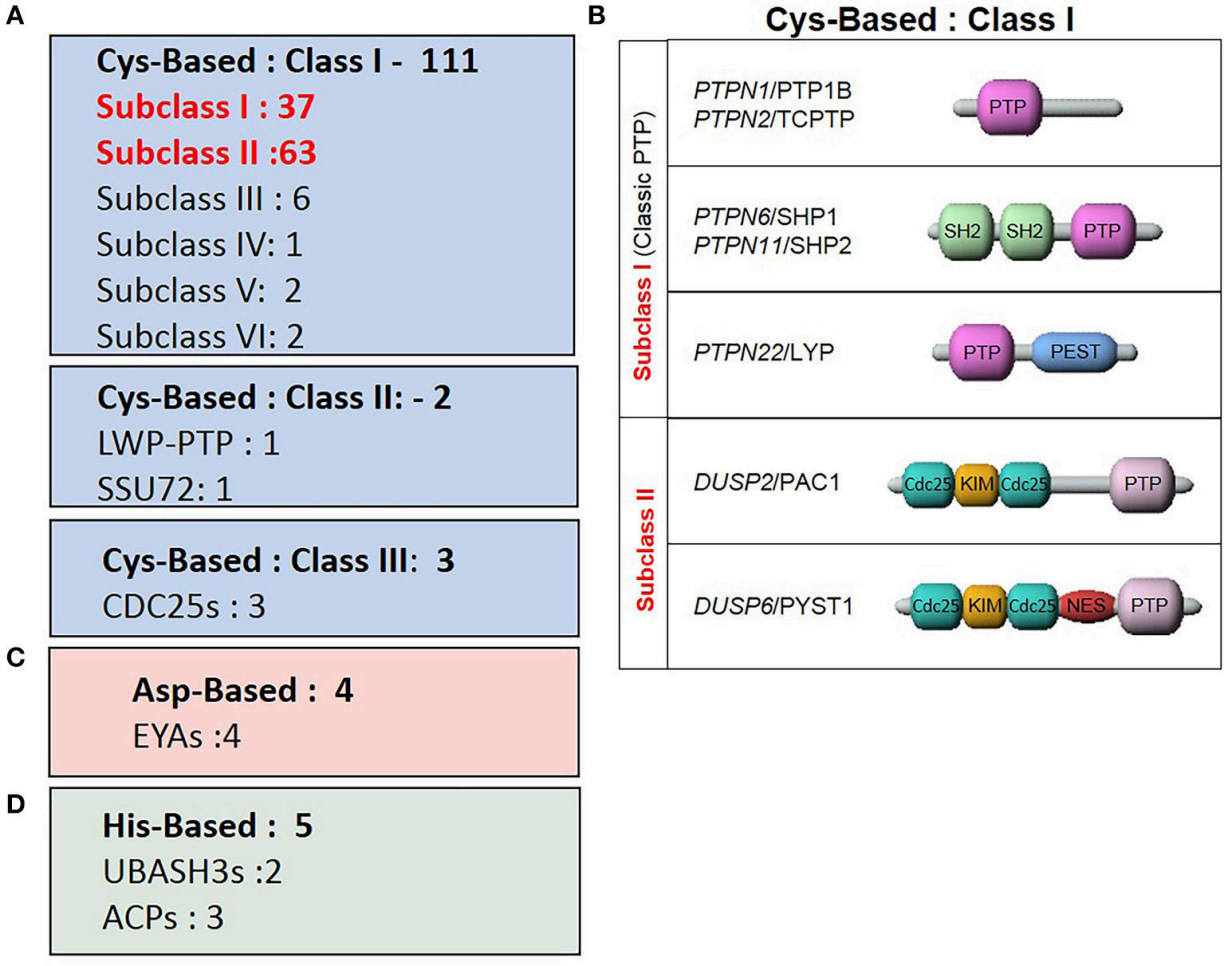
Classes of the tyrosine phosphatase family. **(A)** Sub-classes of Cys-based PTPs and the number of associated genes. **(B)** Representative PTPs from Class I Cys-based enzymes described in the review. **(C)** ASP-based PTPs. **(D)** His-based PTPs.

The human PTP family members are divided into distinct classes based on their structural and biochemical properties (Figure 3). The majority of PTPs have a conserved catalytic domain that contains a cysteine which executes a nucleophilic attack on substrate residues. There are 3 classes of such Cys-based PTPs. Class I is comprised of both classical PTPs that target phosphorylated tyrosine residues, and dual-specific phosphatases (DUSP) that target phosphorylated tyrosine, serine and threonine residues. Class II includes two PTPs, namely low molecular weight PTP and SSU72, whereas Class III comprises the three cell cycle Cdc25 regulatory proteins.

More recent studies characterized enzymes using alternate nucleophilic catalytic residues which were then added to the list of PTP family members. These two additional classes are the aspartic-based and histidine-based acid phosphatase enzymes. Thus, within this classification frame, Alonso and Pulido expanded the PTP gene superfamily to 125 members (66).

Tyrosine phosphorylation is a reversible mechanism that promotes protein–protein interactions and propagates signaling cascades in all cell types. It therefore stands to reason that the PTPs targeting these phosphorylated amino acids play important roles in modulating the strength and duration with which a signaling pathway is activated. Moreover, given that PTPs are a heterogeneous gene family (expression, function, and regulation) it is not surprising that PTPs have been associated with many distinct pathologies in both animal models and in human disease (67). Of importance herein, these include multiple immune-related disorders.

The immune system relies heavily on the use of protein phosphorylation to recognize and transmit intra- and extracellular signals. Signaling cascades initiated at the cell surface by receptor kinases, or from the recruitment of cytoplasmic kinases such as the JAK and Src-like kinase families, translate into multiple immune cell responses like cellular adhesion, division, migration and others. Hence, from the list of over 100 classical Cys-based PTPs, Arimura and Yagi demonstrated that between 58 and 76 of them are expressed in various cell lineages of the immune system (68) and that the vast majority of those (40 to 50) are present in T-cells (69). In T cells, examples of PTP activity mediating both positive and negative regulation of intracellular signaling events have been reported (69).

Multiple genome wide association studies (GWAS) have identified single nucleotide polymorphisms (SNPs) linked with IBD, in or near PTP coding units (70, 71). Examples include the PTPN22, PTPN2, PTPRC, PTPRK, and MTMR3 loci (72). Murine pre-clinical colitis models and tissue-specific PTP knockout mouse strains have been used to discern the role of such PTPs in T cells in the inflamed intestine. Such studies, demonstrated that the perturbation of PTPs such as PTP22, PTPN2, PTPN11, DUSP2, and DUSP6 impacts IBD relevant T cell subsets and/or deregulates T-cell function (73–77). Yet only PTPN22 and PTPN2 have been clearly validated in both patients and animal models for their involvement in IBD.

## PTP regulation of CD4^+^ T cell activity in IBD

### PTPN22

PTPN22 is a non-receptor protein tyrosine phosphatase that is expressed primarily in hematopoietic cells (78). It has been linked with multiple inflammatory disorders and in fact, PTPN22 genetic variation is among the strongest genetic risk factors for autoimmunity diseases such as type I diabetes (T1D), rheumatoid arthritis and systemic lupus erythematosus (79–83). One of the most extensively studied SNPs within the PTPN22 locus is SNP rs33996649 (G788>C) which causes a substitution of arginine 263 with a glutamine residue (263Q variant). While this variant increases susceptibility to multiple autoimmune diseases, it is in fact protective against CD (12, 79). The mechanism mediating this differential effect on disease susceptibility remains unclear. It has been hypothesized that it is due to the different role innate vs. adaptive immune cells play in the onset of these distinct inflammatory diseases (77). Further studies examining the cell type specific effects of the G788>C variant will provide clarity, although the continued debate regarding whether the variant encodes a gain-of-function or altered-function protein will need to be resolved (84–87).

*PTPN22* deficient mice exhibit a lymphoproliferative disease and accumulate memory-phenotype T cells with age, but do not display signs of spontaneous autoimmunity (88). It is important to note that a PTPN22 deficiency can cooperate with the E613R CD45 mutant to induce autoimmune diseases. This suggests that the loss of PTPN22 can play a role in increasing susceptibility to certain inflammatory disorders (89).

A comprehensive examination of the different T cell compartments in *PTPN22*^−/−^ mice provided an example of how cellular context can change the importance of the regulatory function of a given PTP. During the first 2 days following TCR activation, *PTPN22*^+/+^ and *PTPN22*^−/−^ T cells display comparable proliferation, cytokine production and expression of activation markers. Beyond 2 days however, *PTPN22*^−/−^ T cells exhibit an increased rate of cell cycling. In addition, reactivated knockout effector T cells undergo a more rapid and robust proliferation and secrete higher levels of cytokines compared to controls. Likewise *ex vivo* stimulation of CD44^HI^ CD62L^LO^ *PTPN22*^−/−^ effector/memory cells display an increased proliferative response upon stimulation. This has been attributed to enhanced and prolonged phosphorylation of the Lck auto-regulatory (Tyr394) tyrosine following TCR ligation and not due to changes to IL-2 or IL-15 receptor signaling (88).

PTP localization also contributes to the regulation of PTP activity. In addition to Lck, Zap70 has been shown to be a substrate of PTPN22 and both Csk and Vav have been shown to be interacting partners. Csk plays a critical role in sequestering PTPN22 from the TCR complex, thereby limiting the capacity of PTPN22 to inhibit TCR signaling (90, 91–93). The kinetics and localization of PTPN22 activity, and its binding partners in antigen experienced vs. inexperienced T cells, remains unclear. Such an understanding would provide insight into why PTPN22 is particularly important beyond the initial stage of T cell activation.

Murine colitis models indicate that PTPN22 does not solely play a role in the function of conventional T cells. Using the T cell transfer colitis model, PTPN22 was shown to contribute to both effector and regulatory T cells in colitis. The transfer of *PTPN22*^−/−^ naïve CD4^+^CD45RB^HI^ T cells into an immune-deficient recipient resulted in a more severe colitis phenotype, characterized by rapid weight loss and decreased survival. Strikingly, while co-transfer of WT Tregs with *PTPN22*^−/−^ naïve CD4^+^CD45RB^HI^ T cells did not suppress disease, co-transfer of *PTPN22*^−/−^ Tregs did. Protection was attributed to the increased secretion of anti-inflammatory cytokines by *PTPN22*^−/−^ Tregs (76). It then follows that the lack of spontaneous autoimmunity in the *PTPN22*^−/−^ mice may be due to a coincident increase in both effector and regulatory T cell activity.

Further investigation is required to determine if human PTPN22 variants contribute to IBD pathology through their effects on T cell homeostasis in the gut. Although two independent studies confirmed the role of PTPN22 in acute colitis using the dextran-sodium sulfate (DSS) induced model, these studies attributed the increase in disease severity to changes in TLR4 signaling, type I interferon production and macrophage polarization (94, 95).

### PTPN2

PTPN2 is a classical cytoplasmic phosphatase expressed ubiquitously, with the highest levels of expression being detected in lymphoid cells. Its critical role in the homeostasis of the immune system is evident by the progressive systemic inflammatory disease that develops in PTPN2 deficient mice within 1–2 weeks of birth (96). Pro-inflammatory mediators IFNγ, TNFα, and inducible nitric oxide synthase (iNOS) are readily detectable as early as 3 days after birth, followed by the infiltration of mononuclear cells into lymphoid and non-lymphoid organs (97). In comparison, T-cell specific PTPN2 deficient mice do not develop systemic inflammation but rather develop spontaneous autoimmunity at older ages. A reduction in the TCR threshold in both *PTPN2*^−/−^ CD4 and CD8 was identified as causing increased T cell activation due to hyper-phosphorylation of the activating tyrosine residue of Lck (98). It was then proposed that through its negative regulation of TCR signaling, PTPN2 contributes to the maintenance of immune homeostasis.

The loss of PTPN2 has been shown to alter CD4^+^ T cell differentiation, impacting disease severity in multiple murine colitis models. In the T cell transfer colitis model, the transfer of naïve *PTPN2*^−/−^ CD4^+^ T cells resulted in an earlier onset of disease, as well as an increased in weight loss, spleen weight and macroscopic signs of colitis. These clinical signs correlated with more pronounced intestinal inflammation as detected by histological scoring of immune infiltration and epithelial damage. The more pronounced disease severity was confirmed in both acute and chronic DSS colitis models, which both demonstrated a more dramatic weight loss and colonic shortening (99).

Mechanistically, an increase in STAT1 and STAT3 phosphorylation was detected in whole colon lysates. An increase in STAT1/3 phosphorylation was also detected in *PTPN2*^−/−^ CD4^+^ T cells cultured under polarization conditions. The effects of PTPN2 loss on T_H_ differentiation *in vivo* were also examined. In the T cell transfer colitis model, the introduction of PTPN2 deficient CD4^+^ T cells resulted in an almost 3-fold increase in the frequency of IFNγ producers and a 2-fold increase in the frequency of IFNγ^+^ IL17^+^ double producers. In contrast, the frequency of Tregs was reduced over 3-fold. These findings were also recapitulated in the DSS model, although with a less pronounced effect that most likely reflected the fact that pathogenic T cells are not induced robustly in this model (99). One point for further investigation is whether the observed increase in STAT1/3 phosphorylation alters the propensity to commit to a given subset, or if it heightens proliferation rates following commitment.

Importantly, the consequences of PTPN2 loss in murine CD4^+^ T cell differentiation are in line with the sequencing of human PTPN2 variants that have been to be associated with CD. Specifically, transcriptional profiling of CD patients expressing the PTPN2 loss-of-function variant (rs1893217) presented higher expression of T_H_1 and T_H_17-related transcription factors and cytokines (serum and intestinal biopsies) in comparison to PTPN2 expressing CD patients. Such human studies solidify the important role of PTPN2 CD4^+^ T cell responses in the context of human IBD (99).

### PTPN6

The Src homology region 2 domain-containing tyrosine phosphatase-1 (SHP-1, *PTPN6*) is a cytoplasmic phosphatase expressed in all hematopoietic cell lineages throughout development and activation (100–103). Functionally, PTPN6 has been shown to negatively regulate signaling downstream of multiple receptors which co-ordinate immune cell homeostasis and function. These include antigen, cytokine, chemokine and integrin receptors (104–115). As such, it is not surprising that systemic inflammation is the dominant phenotype of the motheaten (*me*) and motheaten viable (*me*^*v*^) mice. These mice harbor mutations that result in undetectable and reduced PTPN6 expression respectively. Similar to PTPN2 deficient mice, *me/me* mice succumb to disease 2–3 weeks following birth (116, 117).

Due to the complex inflammatory pathology of the *me/me* mice, it has proven difficult to dissect the T cell intrinsic effects of a *PTPN6* deficiency. Extensive characterization of T cells derived from *me/me* mice suggests PTPN6 plays a role in regulating the threshold for TCR activation in thymic and peripheral T cells. *Ex vivo* stimulation of *me/me*-derived T cells, as well as T cells expressing a PTPN6 dominant-negative allele, results in a hyper-proliferative response at low antigen concentrations (104, 118–121). Mechanistically, PTPN6 can dephosphorylate multiple proteins downstream of the TCR complex (104, 122–125). Whether one substrate is the physiological substrate, critical to setting the threshold of TCR activation remains a matter of debate.

*In vivo*, the generation of T cell specific PTPN6 deficient mice by different groups produced conflicting findings regarding the role of PTPN6 in T cell polarization. Johnson et al. reported that the loss of PTPN6 does not alter thymocyte or peripheral T cell sensitivity to TCR activation. Rather, the authors note a higher frequency of memory phenotype T cells in the peripheral T cell pool. Given that memory-phenotype T cells respond more robustly upon TCR activation, a hyper-proliferative response is observed when a heterogenous pool of peripheral T cells is stimulated *ex vivo*. The same study demonstrated that a PTPN6 deficiency causes a skewing toward the T_H_2 lineage, associated with sustained IL-4-STAT6 signaling (126). In direct contrast, Martinez et al., also using a T cell specific conditional knockout mouse, showed that PTPN6 depletion lowers the threshold of TCR activation and causes an increase in thymic negative selection and impairs the T cell repertoire (127). Most recently, it has been reported that in an alternate conditional knockout model, in which PTPN6 is deleted in post-selection thymocytes, CD4^+^ T cells are hyper-responsive to TCR stimulation and are intrinsically more resistant to Treg suppression (128).

An understanding of the link between PTPN6 and IBD still needs to be established. For example, a characterization of colitis induction and progression in a PTPN6 T cell conditional knockout mouse has not been published. It remains unknown therefore, whether PTPN6 plays a role in CD4^+^ T cell biology in the inflamed gut. Although limited in scope, two published reports suggest a link between PTPN6 in human colitis though. The levels of PTPN6 were quantified in 98 colonic biopsies, and found to be significantly reduced in active UC, quiescent UC and active CD when compared to healthy controls (129). In a separate study, 2 PTPN6 SNPs (rs7310161 and rs759052) were genotyped in 107 IBD patients and 162 healthy controls from Southern Tunisia (130). A weak association with UC was identified which requires confirmation in a larger cohort. Noteworthy, such studies do not demonstrate that it is the deficiency of PTPN6 in CD4^+^ T cells that is implicated in the disease pathology.

### PTPN11

PTPN11 encodes the Src homology 2-containing protein tyrosine phosphatase 2 (SHP-2). Similar to PTPN6, PTPN11 regulates signaling downstream of multiple surface receptors including growth factor receptors, cytokine receptors and integrins. Its broad tissue expression is in line with the embryonic lethality of PTPN11 knockout mouse at mid-gestation in (131–133).

Extensive human genetic data indicates that PTPN11 has an important role in human disease. Specifically, somatic *PTPN11* mutations in patients with multiple cancer types including leukemias, breast cancer and gastric cancer (134) have been genotyped. As well, in a cohort of 114 Japanese patients, a genetic association has been made between a SNP within the PTPN11 locus and UC (135).

Surprisingly, characterization of T cells from multiple PTPN11 dominant negative knock-in mice and PTPN11 knockout mice, has not lead to a conclusive understanding of this PTP's role in T cell biology. Examples of both deficient- and hyper-T cell activation can be found in these studies. This has been in part attributed to the scaffolding properties of PTPN11 that are retained in the catalytically dead knock-in mouse (73, 136–141). As an example of the scaffolding properties of PTPN11, it has been shown to directly interact with STAT1 and retain STAT1 in the cytoplasm thereby impeding its recruitment to the IFNγ receptor. This disruption of IFNγ signaling, was sufficient to inhibit the production of T_H_1 cytokines and improve 2, 4, 6-trinitrobenzene sulfonic acid induced colitis (142).

By comparison, a PTPN11 conditional T-cell specific knockout mouse has been reported to exhibit increased susceptibility to DSS induced acute colitis. Phenotypic analysis of the T cell compartments in the PTPN11 T cell deficient mice, did not identify any change in the frequency of peripheral T cells as compared to control mice. However, the severity of DSS-induced colitis was found to be much more pronounced in the deficient mice. This increase in severity manifested as a higher body weight loss, disease activity index and colon shortening. It was also reported that an increase in the infiltration of immune cells was observed. Cytokine profiling identified an increase in pro-inflammatory cytokines including IFN-γ, IL-17A, TNF-α, and IL-6 in the mucosa, which correlated with an increased number of T_H_1 and T_H_17 cells in the spleen and lamina propria of T cell specific PTPN11 deficient mice (73).The increases risk of development cancer in UC and CD patients in consistent with the role chronic inflammation plays in tumor initiation and progression. It was therefore surprising that despite the increased inflammation observed in mice harboring a PTPN11 deficiency in CD4^+^ T cells, mice were protected against colitis associated cancer. Specifically, PTPN11 CD4^+^ T cell deficient mice display enhanced T_H_1 immunity and aggravated colitis, but the deficiency inhibited the development of AOM-DSS colitis-associated carcinoma. Mice contained fewer and smaller tumors which expressed reduced levels of proliferation markers. In direct contrast, the progression and metastasis of melanoma was accelerated in the same mouse model. This apparent contradiction suggests that the effects of PTP loss in T cells on tumor progression are highly dependent on the location and stage of the tumor. It must be noted however, that an understanding of how the modulation of PTPs such as SHP-2, impacts the interface between CD4^+^ T cells and tumors, remains largely unexplored (73).

It is important to note that PTPN11's role in maintaining intestinal homeostasis is not restricted to CD4^+^ T cells. In fact, an intestinal epithelial specific PTPN11 knockout mouse has been generated and develops severe colitis. Such mice have a severe defect in the intestinal barrier which is associated with impaired tight junction formation, goblet cell differentiation and IEC migration (143, 144).

### DUSP2

DUSP2 is a mitogen- and stress-inducible nuclear DUSP that is enriched in lymphoid tissue and differentially expressed in CD4^+^ T cell subsets. Possibly due to compensation by other DUSP family members, DUSP2 deficient mice are viable and exhibit no gross defects and no alterations in the frequency or absolute number of lymphoid populations in the thymus, spleen or lymph nodes. However, using *in vitro* models of T cell polarization, it was demonstrated that in the absence of DUSP2, T_H_17 cell differentiation is promoted while inducible Treg (iTreg) differentiation is suppressed (74).

To question whether such modulation of *in vitro* T_H_ differentiation relates to effects observed *in vivo*, two mouse models of intestinal inflammation were used. First, in acute and chronic DSS colitis models, *Dusp2*^−/−^ mice exhibited a more severe disease as assessed by weight loss, clinical scoring, survival and histopathology scoring. Higher colonic inflammation was attributed to an increased proportion of colonic Il-17A^+^ CD4^+^ T cells in *Dusp2*^−/−^ mice as compared to control mice, whereas T_H_1 and Treg populations appeared to be unaltered in DSS treated mice. Second, a T cell transfer colitis model has confirmed the T cell intrinsic effects of a DUSP2 deficiency. To do so, severe combined immunodeficiency (SCID) mice were reconstituted with either WT or *DUSP2*^−/−^ CD4^+^CD45RB^hi^ T cells. In this model, an increase in body weight loss, severity of histopathology scoring, and colon thickening were observed following the transfer of *DUSP2*^−/−^ T cells compared to controls. Such clinical signs were corroborated by a rise in the number of colon infiltrating CD4^+^ T cells expressing a T_H_17 gene signature. Moreover, such T_H_17 T cells in the inflamed tissue expressed elevated levels of pathogenic T_H_17-associated genes but not non-pathogenic genes (74).

Given evidence that Treg differentiation is suppressed in *in vitro* differentiation assays, the ability of *DUSP2*^−/−^ Tregs to suppress colitis in the T cell transfer model was evaluated. Surprisingly, DUSP2 was found to be dispensable, as *DUSP2*^−/−^ Tregs were as efficient as WT Tregs in suppressing inflammation when co-transferred with WT CD4^+^CD45RB^hi^ cells into SCID mice (74).

To identify the molecular mechanism by which DUSP2 limits T_H_17 differentiation, phosphoproteomic analysis was performed on colon homogenates from DSS treated WT and *DUSP2*^−/−^ mice. Along with follow-up biochemical studies, STAT3 was identified as a direct substrate of DUSP2. DUSP2 was shown to dephosphorylate residues Tyr705 and Ser727. Although comparable levels of basal colonic STAT3 phosphorylation were reported between WT and *DUSP2*^−/−^ mice, the levels were higher in *DUSP2*^−/−^ DSS-challenged mice compared to WT DSS-challenged mice (74). STAT3 is critical in driving disease in T-cell transfer models of colitis (145), the authors propose that the *DUSP2*^−/−^ phenotype is due to elevated STAT3 activity. It should be noted however, that the authors have not formally proven that the phenotype is dependent on STAT3 activity.

DUSP2 has also been implicated in human IBD. The expression of DUPS2 mRNA is reduced in the peripheral blood of UC patients (*n* = 24) and is only increased minimally following PMA stimulation. The low levels of DUPS2 expression were found to be attributed to CpG methylation. Unfortunately, the levels of DUSP2 in colonic biopsies, or colonic T cells, were not quantified. As such, further evidence is required to definitely demonstrate that a reduction in T cell DUSP2 occurs in IBD patients and that this reduction impacts human T cell polarization in the disease state (74).

Based on these findings it has been proposed that under healthy conditions, T cell activation results in a transient increase in DUSP2 which reduces STAT3 activation below levels required to promote T_H_17 differentiation. In the absence of DUSP2, elevated levels of STAT3 phosphorylation result in the differentiation of pathogenic T_H_17 cells which promote inflammation (74).

### DUSP6

Extracellular signal-regulated kinase (ERK) signaling regulates multiple cellular responses to activation including T cell proliferation, cytokine production, survival and adhesion. CD4^+^ T cell polarization *in vivo* has also been shown to be modulated by ERK signaling, numerous reports suggesting ERK activity impacts T_H_1 and T_H_2 differentiation and the T_H_17:Treg balance. (146–153).

DUSP6 is a cytoplasmic ERK-specific DUSP (154, 155). Surprisingly, genetic ablation of DUSP6 does not cause developmental defects despite an increase in the basal levels of ERK phosphorylation in the heart, spleen, kidney, and brain. Rather, DUSP6 deficient mice are viable with no reported phenotype in the steady state (156). Initial cellular *in vitro* assays suggested a role for DUSP6 in the regulation of CD4^+^ T cell activation. Specifically, DUSP6 was shown to be upregulated following TLR4 stimulation and then restrained ERK activation and suppressed IFNγ production by TCR stimulation (157).

More recently, a more in depth characterization of DUSP6 in CD4^+^ T cells has been reported (75). *DUSP6*^−/−^ mice exhibited no change in the frequency or absolute number of peripheral CD4^+^ or CD8^+^ T cells. Nevertheless, a higher percentage of memory-effector CD4^+^ T cells and a lower percentage of naïve CD4^+^ T cells in *DUSP6*^−/−^ mice, indicates an increase in CD4^+^ T cell activation. *Ex vivo* TCR-stimulated of *DUSP6*^−/−^ CD4^+^ T cells display increased ERK activation and proliferation but also an elevated rate of activation induced cell death. Polarization studies demonstrated that DUSP6 depletion promotes T_H_1 differentiation and increases IFNγ production, whereas expression levels of IL-2, IL-4, IL-6, or IL-10 are not altered. In contrast T_H_17 CD4^+^ T cells displayed decreased survival and IL-17A secretion, leading to the conclusion that DUSP6 suppresses the T_H_1 lineage and promotes T_H_17 differentiation (75).

The role of DUSP6 in intestinal inflammation was addressed using an *IL-10*^−/−^ colitis model. *IL-10*^−/−^*DUSP6*^−/−^ double knockout mice were generated and phenotyped. Such mice had accelerated and exacerbated colitis. Histological examination identified elevated epithelial crypt hyperplasia, goblet-cell depletion, and infiltration of mononuclear cells. Moreover, colonic explants were found to secrete higher levels of TNFα and IFNγ, while IL-17A levels were reduced. Strikingly, ERK inhibition was shown to significantly reduce colitis in *IL-10*^−/−^*DUSP6*^−/−^ mice, both prophylactically and therapeutically. The severity of both crypt hyperplasia and immune cell infiltration was reduced. Despite, these intriguing findings, future studies are needed to demonstrate that the protective role of DUSP6 in colitis is intrinsic to CD4^+^ T cells (75).

## PTPs as therapeutic targets for IBD?

In the late 1990s, multiple research programs sought to identify the PTP(s) that recognize and dephosphorylate the insulin receptor (IR). The regulation of IR activation by a PTP was hypothesized to decrease IR mediated signaling events, and subsequent entry of glucose into insulin receptive tissues. It was believed that a rise in the expression or activity of such a regulatory PTP would result in high levels of blood glucose in the circulation, which would then instigate type II diabetes. This hypothesis was confirmed by two seminal publications which described the PTP1B knockout mouse that presented with an increase in IR phosphorylation. These studies validated PTP1B as an outstanding candidate to be targeted for the treatment of type II diabetes (158, 159) and led to major efforts to identify small inhibitors of PTP1B. Indeed, since their publications these reports were cited over 2500 times, primarily in the context of depicting the isolation of novel PTP1B inhibitors.

In spite of all these reports, there are no small molecule inhibitors against PTP1B (or any PTP) that have been successfully developed beyond initial clinical trials. Multiple reviews have already examined the difficulties associated with developing PTP competitive inhibitors and this subject is beyond the scope of this review (160–163). In brief, the main challenges in developing inhibitors are the high polarity and homology of their catalytic pockets. Hence, chemical screens for competitive small molecule PTP inhibitors, have only isolated inhibitors with poor cell permeability and low specificity. Excitement has been garnered by the recent development of allosteric PTP inhibitors such as the PTP1B carboxyl domain compound (164) and a PTPN11 inhibitor from Novartis (165) which may lead to the development of novel pharmaceuticals.

It is worthy to note that novel therapeutic strategies are also being developed to target PTPs. These include the forced dimerization and inhibition of receptors PTPs (166, 167), intrabodies inhibitors (168), small molecular caged compound inhibitors (169) and even RNA aptamers (170) that modulate the enzymatic activity of PTPs. As well, genetic modulation through CRISPR technologies (171), as well as protein degradation technologies (PDTs) such as PROTACs (PROteolysis TArgeting Chimeras) (172) and SNIPERS (Specific and Non-genetic IAP-dependent Protein Erasers) (173) are now exciting avenues for tackling the difficult PTP gene family.

Significant effort is also being made in the development of cell based therapies. Potential adverse effects that could be associated with systemically inhibiting PTPs are mitigated by using PTP inhibitors in *ex-vivo* cell cultures. This allows for the modulation of PTP activity in a cell-type specific and temporary fashion. For example, we reported a protocol for dendritic cell (DC) vaccination that employs inhibitors of PTPN1-PTPN2 (174). This work demonstrated that in the proper context, PTP inhibitors may have broad application in cancer and infectious diseases. Beyond DCs, other cell types such as macrophages, natural killer cells, and of course T-cells as described above, may also be malleable by PTP inhibition and useful in various cell therapies.

From an IBD perspective, it is clear that inhibiting the PTPs presented above would most likely exacerbate disease by potentiating the effects of pro-inflammatory cytokines and promoting the differentiation of pathogenic T cells. Indeed this contention is supported by the fact that, biologics and small molecules that suppress cytokine receptor signaling have been clinically successful in IBD disease management. One example would be tofacitinib, a JAK inhibitor found to be effective in phase 2 and 3 trials in moderate to severe ulcerative colitis (175).

Our understanding of PTP function in immune cells is expanding beyond cytokine receptor signaling, and our capacity to modulate cellular responses by titrating PTP expression is also evolving at a rapid pace. The cell-dependent context of positive or/and negative modulation bestowed by the nearly 80 PTPs expressed in immune cells, remains an exciting ground for study and clinical improvement. It remains to be seen if this renewed interest in PTP inhibitors would be applicable to inflammatory diseases such as UC and CD.

## Author contributions

All authors listed have made a substantial, direct and intellectual contribution to the work, and approved it for publication.

### Conflict of interest statement

The authors declare that the research was conducted in the absence of any commercial or financial relationships that could be construed as a potential conflict of interest.
